# Polymorphisms in the Presumptive Promoter Region of the SLC2A9 Gene Are Associated with Gout in a Chinese Male Population

**DOI:** 10.1371/journal.pone.0024561

**Published:** 2012-02-29

**Authors:** Changgui Li, Nan Chu, Binbin Wang, Jing Wang, Jian Luan, Lin Han, Dongmei Meng, Yunlong Wang, Peisu Suo, Longfei Cheng, Xu Ma, Zhimin Miao, Shiguo Liu

**Affiliations:** 1 The Affiliated Hospital of Medical College, Qingdao University, Qingdao, China; 2 Graduate school, Peking Union Medical College, Beijing, China; 3 National Research Institute for Family Planning, Beijing, China; 4 Qingdao Municipal Hospital, Qingdao, China; 5 World Health Organization Collaborating Centre for Research in Human Reproduction, Beijing, China; Mayo Clinic College of Medicine, United States of America

## Abstract

**Background:**

Glucose transporter 9 (*GLUT9*) is a high-capacity/low-affinity urate transporter. To date, several recent genome-wide association studies (GWAS) and follow-up studies have identified genetic variants of SLC2A9 associated with urate concentrations and susceptibility to gout. We therefore investigated associations between gout and polymorphisms and haplotypes in the presumptive promoter region of *GLUT9* in Chinese males.

**Methodology/Principal Findings:**

The approximately 2000 bp presumptive promoter region upstream of the start site of exon 1 of *GLUT9* was sequenced and subjected to genetic analysis. A genotype-phenotype correlation was performed and polymorphisms-induced changes in transcription factor binding sites were predicted. Of 21 SNPs identified in *GLUT9*, five had not been previously reported. Two of the SNPs (rs13124007 and rs6850166) were associated with susceptibility to gout (p = 0.009 and p = 0.042, respectively). The C allele of rs13124007 appeared to be the risk allele for predisposition to gout (p = 0.006, OR 1.709 [95% CI 1.162–2.514]). For rs6850166, an increased risk of gout was associated with the A allele (p = 0.029, OR 1.645 [95% CI 1.050–2.577]). After Bonferroni correction, there was statistically difference in rs13124007 allele frequencies between gout cases and controls (*P* = 0.042). Haplotype analyses showed that haplotype GG was a protective haplotype (p = 0.0053) and haplotype CA was associated with increased risk of gout (p = 0.0326). Genotype-phenotype analysis among gout patients revealed an association of rs13124007 with serum triglycerides levels (P = 0.001). The C to G substitution in polymorphism rs13124007 resulted in a loss of a binding site for transcription factor interferon regulatory factor 1 (IRF-1).

**Conclusions/Significance:**

Polymorphisms rs13124007 and rs6850166 are associated with susceptibility to gout in Chinese males.

## Introduction

Gout is a common inflammatory arthritis affecting men, and occurs in 1.94% of the male population residing in the coastal area of Shandong, China [1]. Its clinical features include the presence of elevated serum urate, crystal-induced inflammatory arthritis, painful tophi, uric acid urolithiasis, kidney disease, and metabolic syndrome [2]. The risk factors of gout are complicated due to dietary habits and life-style changes, such as increased alcohol consumption and overindulgence in meat and seafood [1]. Additionally, insulin resistance, abdominal obesity, dyslipidemia, arterial hypertension, diabetes, and the metabolic syndrome are highly associated with hyperuricemia and gout [3], [4]. However, hyperuricemia has long been recognized to be the most important risk factor for gout [5].

In the majority of patients with gout, hyperuricemia occurs as a result of overproduction of uric acid (UA) or impaired excretion of renal uric acid [6], [7]. The mechanisms of urate transport in the kidney is complex, and specialized molecules expressed in renal proximal tubule cells mediate this renal exchange [8]. GLUT9 (SLC2A9) has recently been shown to function as a high-capacity urate transporter and to influences serum uric acid levels [9], [10], [11].

Solute carrier family 2, member 9 (SLC2A9), also known as glucose transporter 9 (GLUT9), is thought to be an important urate transporter belonging to the SLC2A facilitative glucose transporter family. GLUT9 is encoded by the SLC2A9 gene (4p16.1), which contains 13 coding exons and can encode two transcripts, a long isoform (GLUT9L/SLC2A9L) and a short isoform (GLUT9S/SLC2A9S). SLC2A9L and SLC2A9S show strong expression in the basolateral and apical membranes, respectively, of proximal renal tubular cells [12]. Furthermore, they are expressed in the chondrocytes of human articular cartilage, which plays a role in the development of tophaceous gout [13]. The remarkable aspect of GLUT9 function is that it is an important modulator responsible for the reabsorption of urate in the apical membrane of the renal proximal tubules [10]. Consistent with this, loss-of-function mutations in the GLUT9 gene, causing functional impairment, are associated with renal hypouricemia in Japanese [14] and Israeli-Arab [15] patients.

The SLC2A9 gene has received considerable attention and many polymorphisms within or near the gene have been reported. Several recent genome-wide association studies (GWAS) and follow-up studies identified that genetic variants of SLC2A9 had a role in affecting urate concentrations [10], [11], [16], [17], [18], [19], [20], [21], [22], [23], [24] and susceptibility to gout [10], [16], [25], [26], [27], [28], [29], [30]. To the best of our knowledge, these studies mainly focused on exonic and intronic SNPs of the GLUT9 gene. So far, only limited data is available, including a study on Sardinia and Chianti Cohorts, which reported that no variant influencing serum uric acid levels was observed in a 1.5 kb presumptive promoter region upstream of the transcription start site [17]. Therefore, no study to date has evaluated the association between gout and polymorphisms and haplotypes in the presumptive promoter region upstream of the transcription start site. As a result, we initiated a genotype-phenotype analysis of 300 male gout patients and 318 normal male controls, investigating genetic variants in the presumptive promoter region of *GLUT9* associated with gout and their potential phenotypic consequences.

## Methods

### Ethics statement

The study protocol conforms to the ethical guidelines of the 1975 Declaration of Helsinki and informed consent was obtained.

### Study population/phenotyping

A total of 300 male gout patients, who visited the Department of Endocrinology or gout laboratory at the affiliated hospital of the Qingdao University Medical College, were recruited. All the gout patients were diagnosed by a clinical endocrine physician according to the criteria from the American College of Rheumatology in 1977 [31]. Likewise, 318 normal male controls without a personal or familial history of hyperuricemia or gout or other serious illness were collected. All patients and controls were selected from the same population residing in the coastal area of Shandong province, China. Informed consent was obtained from each participant. We measured all the participants for blood glucose, uric acid, total cholesterol (TC), triglycerides (TG), urea nitrogen, and creatinine in the plasma. Phenotypic characteristics included demographic data and clinical parameters (tophi and disease-related complications), which were recorded using a standardized questionnaire. The visible or palpable tophi were determined from patients' arms, legs, knees, ears, olecranon processes, articular cartilage, and other sites.

### PCR amplification/Genotyping

From all study participants, blood samples were taken and genomic DNA was isolated from peripheral blood leukocytes by conventional methods. An over 2000 bp presumptive promoter region upstream of the start site of exon 1 of *GLUT9* was screened by polymerase chain reaction (PCR) and subsequent direct sequencing. The detailed list of primers and PCR conditions can be found in Table S1.

### Statistical analysis

In this study, the Statistical Package for Social Sciences version 16.0 (SPSS Inc., Chicago, IL, U.S.A.) was used for statistical analysis and Haploview 4.2 was used for haplotype frequencies estimation. Student's t-test was used to assess a significant difference in demographic and clinical characteristics between cases and controls. Each genetic marker complied with Hardy-Weinberg equilibrium (HWE) (p>0.01) in the control population. The odds ratios (ORs) and 95% confidence intervals (95% CI) were used as a measure of the strength of relationships in the genotype distribution and allele frequencies of presumptive promoter region between the patient cases and controls, and between tophi patients and non-tophi patients. The P-value was tested by Pearson's chi-square test. Haploview 4.2 was used to calculate linkage disequilibrium blocks and haplotype association risk [32]. An analysis of variance (ANOVA) was used to calculate the association between genotypes and demographic and clinical characteristics among gout patients, including age at diagnosis, duration of gout history, Body Mass Index (BMI), Waist-to-Hip Ratio (WHR), tophi, hypertension, diabetes, obesity, and serum biochemistry. *P* values less than 0.05 were regarded as statistically significant.

### Prediction of presumptive transcription factor binding sites

Changes in transcription factor binding sites caused by nucleotide alterations in the promoter region was performed with AliBaba software (version 2.1)

## Results

### Demographic and clinical characteristics of the study population

The clinical characteristics of the population enrolled in the study are summarized in Table 1. The results showed that gout patients had significantly higher BMI values, blood glucose levels, TC levels, TG levels, uric acid levels, systolic pressure levels, diastolic pressure levels than the controls (*P*<0.001). They also presented significantly lower serum creatinine levels (P = 0.035) when compared with controls. In addition, the mean age (P = 0.938) and serum urea nitrogen levels (P = 0.064) tended to be higher in the gout patients, but did not reach significance. Twenty-eight percent of the patients with gout had tophi.

**Table 1:** Demographic and clinical characteristics (Mean±SD) of the study population.

Parameter	Gout (n = 300)	Control (n = 318)	P
Age (yr)	52.57±13.96	52.47±18.35	*P* = 0.938
Tophi, n (%)	84 (28%)	—	—
BMI (kg/m^2^)	27.11±3.10	23.63±3.29	*P*<0.001
Systolic pressure(mmHg)	137.4±19.8	128.6±18.8	*P*<0.001
Diastolic pressure(mmHg)	88.4±12.2	79.4±10.3	*P*<0.001
Blood Glucose (mmol/L)	6.21±2.06	5.22±0.90	*P*<0.001
Uric acid (umo1/L)	515.05±140.95	322.24±53.31	*P*<0.001
Triglycerides (mmol/L)	2.28±1.75	1.06±0.54	*P*<0.001
Total cholesterol (mmol/L)	5.25±1.33	4.79±0.86	*P*<0.001
Urea nitrogen (mmol/L)	6.10±3.78	5.66±1.44	*P = 0.064*
Creatinine (umo1/L)	92.77±39.68	97.78±11.15	*P* = 0.035

### Genetic analyses

The population enrolled in the study was age-matched. To determine the potential genetic variants in the promoter of *GLUT9*, we sequenced over 2000 bp presumptive promoter region upstream of the start site of exon 1 of *GLUT9*. Through these studies, we identified and confirmed 21 single nucleotide polymorphisms (SNPs) (Table 2). Among these, we identified five novel polymorphisms that had not been previously reported; the remaining 16 SNPs had already been deposited in the National Center for Biotechnology Information SNP database (NCBI dbSNP).

**Table 2:** Genotype distribution and allele frequencies of promoter of the human GLUT9 gene in cases and controls.

		Genotype frequency of cases#			Genotype frequency of controls#			Genotype frequency			Minor Allele Frequencies			
dbSNP ID(allele 1/allele 2)#	position	1/1	1/2	2/2	1/1	1/2	2/2	χ^2^	P value	Minor Allele	case	control	χ^2^	P value
rs13124007(G/C)	10043931	233	62	5	271	47	0	9.413	0.009	C	0.120	0.074	7.541	0.006
rs62293415(T/G)	10043886	84	150	66	88	146	84	1.784	0.41	G	0.470	0.494	0.695	0.4044
New1*(G/A)	10043821	296	4	0	315	3	0	0.21	0.647	A	0.007	0.005	0.208	0.648
New2*(A/G)	10043790	294	4	2	312	6	0	2.412	0.299	G	0.013	0.009	0.419	0.5173
rs77678083(G/A)	10043724	296	4	0	311	7	0	0.665	0.415	A	0.007	0.011	0.659	0.4169
rs6850166(G/A)	10043688	253	43	4	284	34	0	6.323	0.042	A	0.085	0.053	4.796	0.0285
New3*(G/A)	10043648	292	8	0	311	7	0	0.141	0.707	A	0.013	0.011	0.139	0.7088
rs36036984(G/A)	10043632	133	119	48	128	130	60	1.392	0.499	A	0.358	0.393	1.589	0.2075
rs78201117(G/A)	10043165	293	7	0	310	8	0	0.022	0.883	A	0.012	0.013	0.021	0.8836
rs13137343(G/T)	10043028	84	152	64	101	136	81	3.923	0.141	T	0.467	0.469	0.004	0.947
rs79763149(G/A)	10042974	285	15	0	296	21	1	1.685	0.431	A	0.025	0.036	1.291	0.2558
rs13101785(A/T)	10042915	85	150	65	88	145	85	2.281	0.32	T	0.467	0.495	1.013	0.3143
rs7685958(G/C)	10042850	105	149	46	107	148	63	2.151	0.341	C	0.402	0.431	1.079	0.2988
rs13137074(G/A)	10042842	89	148	63	88	147	83	2.226	0.329	A	0.457	0.492	1.558	0.212
rs61476037(G/A)	10042673	276	24	0	304	14	0	3.462	0.063	A	0.040	0.022	3.352	0.0671
rs7349721(A/T)	10042562	101	147	52	104	144	70	2.208	0.332	T	0.418	0.447	1.001	0.3172
rs61446121(G/A)	10042242	281	19	0	289	29	0	1.673	0.196	A	0.032	0.046	1.605	0.2052
New4*(G/A)	10042221	298	2	0	313	5	0	1.131	0.288	A	0.003	0.008	1.124	0.289
rs7679916(G/A)	10042160	79	158	63	92	144	82	3.606	0.165	A	0.473	0.484	0.148	0.7003
rs10516197(G/A)	10042100	103	153	44	108	147	63	3.091	0.213	A	0.402	0.429	0.967	0.3255
New5*(G/A)	10042027	288	12	0	305	13	0	0.003	0.956	A	0.020	0.020	0.003	0.9562

* Novel SNP not reported in public database before.

# The major allele was referred to as allele 1 and the minor allele as allele 2.

All 21 SNPs followed the Hardy–Weinberg equilibrium (HWE) in the control group (cutoff p-value = 0.01). Table 2 shows the genotype frequencies in cases and controls. Allelic frequency distributions of the two promoter SNPs showed statistical significance of p = 0.006 and p = 0.029. For rs13124007, a G/C polymorphism, the C allele seemed to be the risk allele for predisposition to gout. (p = 0.006, OR 1.709 [95% CI 1.162–2.514]) (Table 3). While for rs6850166, a G/A polymorphism, an increased risk of gout was associated with the A allele (p = 0.029, OR 1.645 [95% CI 1.050–2.577]) (Table 3). For all other SNPs investigated, we did not reveal significant differences in allele frequencies between the gout patients and the controls (Table 2). In accordance with the allelic associations, the genotype distribution differed significantly between these two groups for two promoter SNPs rs13124007 and rs6850166 (p = 0.009 and p = 0.042 for respectively). The minor allele frequencies of SNPs containing New1, New2, rs77678083, New3, rs78201117, rs79763149, rs61476037, rs61446121, New4 and New5 were very low (<5%) and the 10 polymorphisms were not participate in calculating. Besides, the linkage disequilibrium (LD) measure r^2^ was estimated and 5 polymorphisms rs62293415, rs13137343, rs13101785, rs13137074, and rs7679916 which were in high LD with each other (*r* ^2^>0.9) were selected to represent a block. Then *P* values multiplied the block together with the remaining 6 polymorphisms containing rs13124007, rs6850166, rs36036984, rs7685958, rs7349721 and rs10516197 which participate in Bonferroni correction. The final modified P values less than 0.05 were regarded as statistically significant. After Bonferroni correction, there was statistically difference in rs13124007 allele frequencies between gout cases and controls (*P* = 0.042).

**Table 3:** The associations in the distributions of genotypes and allele frequency for two polymorphisms (rs13124007 and rs6850166) in the GLUT9 gene in cases and controls.

	(1)	(2)	(1+2)	(3)	(1)vs.(2)	(1)vs.(3)	(1+2)vs.(3)
	Tophi patients	Non-tophi patients	Gout patients	Control	p value	p value	p value
	(n = 84)	(n = 216)	(n = 300)	(n = 318)	OR(95% CI)	OR(95% CI)	OR(95% CI)
Polymorphism rs13124007							
Genotypes							
GG	60	173	233	271			
GC	23	39	62	47	0.193	0.003	0.009
CC	1	4	5	0			
Alleles							
G	143	385	528	589	0.176	0.002	0.006
C	25	47	72	47	1.432(0.850–2.413)	2.191(1.305–3.679)	1.709(1.162–2.514)
Polymorphism rs6850166							
Genotypes							
GG	69	184	253	284			
GA	14	29	43	34	0.768	0.046	0.042
AA	1	3	4	0			
Alleles							
G	152	397	549	602	0.575	0.046	0.0285
A	16	35	51	34	1.194(0.642–2.220)	1.864(1.002–3.466)	1.645(1.050–2.577)

Table 3 lists the association of polymorphisms rs13124007 and rs6850166 in genotypes and allele frequency with gout/tophi between cases and controls. We subdivided the gout patients into tophi and non-tophi cases to reveal the distributions of genotypes and allele frequency between tophi patients and non-tophi patients, as well as between tophi patients and controls. The two SNPs were not crucial risk factors for tophus occurrence. Concerning tophi patients and non-tophi patients, the distributions of the genotypes and allele frequencies showed no significant differences (all P>0.05).

**Figure 1:**
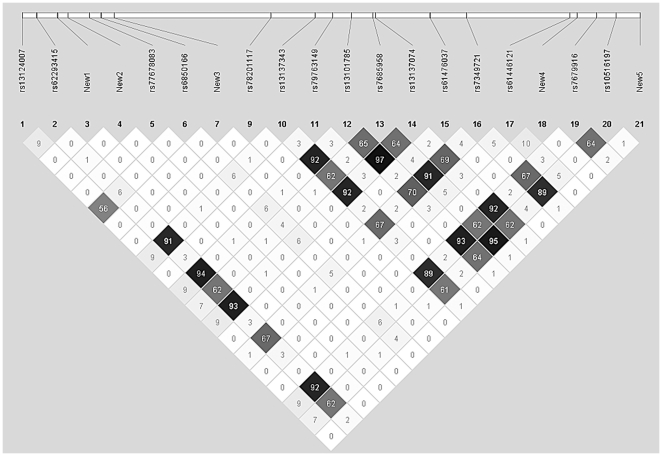
The haplotype association among all 21 SNPs in the GLUT9 gene.

To further investigate the haplotype association among all 21 SNPs in the GLUT9 gene, the linkage disequilibrium (LD) measure r^2^ was estimated using Haploview4.2 software (Figure 1). Two polymorphisms rs13124007 and rs6850166, which were significantly associated with gout, were in low LD (*r* ^2^ = 0.56). Notably, these two polymorphisms defined two major haplotypes, namely the GG and CA haplotypes, representing >95% of all haplotypes. The frequencies of GG, CA, GA and CG haplotypes were 87.3%, 7.8%, 4.2% and 0.7%, respectively, among gout patients, and 92.1%, 4.9%, 2.5% and 0.5%, respectively, among the controls. The GG haplotype was less frequently present in cases than in controls, and could be regarded as a protective haplotype (p = 0.0053, OR 0.588 [95% CI 0.404–0.857]). The CA haplotype was associated with an increased risk for gout (p = 0.0326, OR 1.659 [95% CI 1.039–2.648]).

### Genotype-phenotype analysis

Based on the significant association of the polymorphisms rs13124007 and rs6850166 with the development of gout, an ANOVA test showed a detailed genotype-phenotype analysis among gout patients (Table 4). For polymorphism rs13124007, serum triglycerides levels showed a significantly different distribution among the different genotypes (P = 0.001). Patients carrying genotype CC showed higher triglycerides levels compared to those carrying genotype GG (5.11±3.29 mmol/L vs. 2.28±1.73 mmol/L; *P* = 3.04×10^−4^) and to heterozygous patients (5.11±3.29 mmol/L vs. 2.04±1.5 mmol/L; *P* = 1.38×10^−4^) (Table 4). For polymorphism rs6850166, among gout patients, a significant interaction was found between genotypes and BMI values (*P* = 0.021). In addition, we found that patients with the AA genotype showed significantly higher systolic pressure levels, compared with the GG genotype (*P* = 0.043) and the GA genotype (*P* = 0.046). Moreover, we observed that diastolic pressure levels differed significantly among the three genotypes (p = 0.046), and patients with the GA genotype showed significantly lower diastolic pressure levels, compared with the GG genotype (*P* = 0.048) and the AA genotype (*P* = 0.047). In addition and similar to polymorphism rs13124007, patients with the AA genotype for rs6850166 were found to have significantly higher serum triglycerides levels compared to those with the GA genotype (4.03±1.6 mmol/L vs. 2.19±2.03 mmol/L; *P* = 0.045) or GG genotype (4.03±1.6 mmol/L vs. 2.26±1.69 mmol/L; *P* = 0.045) (Table 4).

**Table 4:** Association between the polymorphisms (rs13124007 and rs6850166) and characteristics among gout patients.

dbSNP ID	(1)1/1*	(2)1/2*	(3)2/2*	(1)vs.(2)vs.(3)	(1)vs.(2)	(1)vs.(3)	(2)vs.(3)	(1)vs.(2+3)	(1+2)vs.(3)
(allele 1/allele 2)*	(n)	(n)	(n)	p value	p value	p value	p value	p value	p value
				OR(95% CI)	OR(95% CI)	OR(95% CI)	OR(95% CI)	OR(95% CI)	OR(95% CI)
rs13124007(G/C)	233	62	5						
Demographic characteristics (Mean ± SD)									
Age (yr)	52.1±14.09	54.81±13.46	46.6±12.07	0.251	0.175	0.383	0.206	0.28	0.336
Age at diagnosis (yr)	45.8±14.01	47.34±13.7	42.6±9.4	0.633	0.44	0.61	0.464	0.539	0.574
Disease duration (yr)	6.28±6.48	7.47±6.75	4±3.67	0.306	0.202	0.439	0.253	0.076	0.39
BMI (kg/m^2^)	27.24±3.08	26.66±3.13	27.18±3.92	0.434	0.197	0.969	0.719	0.304	0.962
WHR	0.92±0.05	0.92±0.05	0.89±0.03	0.621	0.967	0.332	0.339	0.844	0.329
Serum Biochemistry (Mean ± SD)									
Systolic pressure (mmHg)	137±19.05	138.15±22.51	150±18.71	0.333	0.685	0.147	0.199	0.5	0.154
Diastolic pressure (mmHg)	88.78±12.19	86.56±12.08	96±8.94	0.167	0.203	0.189	0.095	0.372	0.161
Blood Glucose (mmol/L)	6.27±2.23	5.95±1.26	6.67±1.76	0.486	0.275	0.671	0.454	0.347	0.618
Uric acid (umo1/L)	509.91±137.79	536.72±141.09	485.6±265.23	0.37	0.184	0.703	0.436	0.24	0.813
Triglycerides (mmol/L)	2.28±1.73	2.04±1.5	5.11±3.29	0.001	0.316	3.04×10−4	1.38×10−4	0.946	0.122
Total cholesterol (mmol/L)	5.31±1.41	4.98±0.97	6.21±0.85	0.06	0.083	0.13	0.045	0.201	0.104
Urea nitrogen (mmol/L)	6.16±4.1	5.95±2.43	4.9±0.87	0.723	0.705	0.643	0.551	0.589	0.477
Creatinine (umo1/L)	93.43±41.03	92.44±35.21	66.12±15.67	0.314	0.862	0.129	0.154	0.593	0.13
Clinical characteristics									
Age at diagnosis (yr)									
<25	11/233	1/62	0	0.486	0.271	0.619	0.775	0.235	0.645
					0.33(0.04–2.61)			0.33(0.04–2.61)	
25–44	109/233	24/62	2/5	0.512	0.256	0.764	0.955	0.248	0.821
					0.72(0.41–1.27)	1.71(0.28–10.40)	2.38(0.37–15.27)	1.04(0.60–1.79)	2.42(0.40–14.68)
45–64	84/233	29/62	3/5	0.187	0.123	0.271	0.569	0.083	0.323
					1.56(0.89–2.75)	2.66(0.44–16.24)	1.71(0.27–10.94)	1.62(0.94–2.81)	2.42(0.40–14.68)
65–84	29/233	8/62	0	0.696	0.923	0.4	0.392	0.912	0.398
					1.04(0.45–2.41)			1.04(0.45–2.41)	
Tophi	60/233	23/62	1/5	0.193	0.077	0.771	0.443	0.106	0.688
					1.70(0.94–3.08)	0.72(0.08–6.58)	0.42(0.05–4.03)	2.10(1.15–3.83)	0.64(0.07–5.80)
Past history									
Hypertension#	161/233	40/62	5/5	0.247	0.491	0.137	0.104	0.764	0.128
					0.81(0.45–1.47)	0.69(0.63–0.75)	0.64(0.54–0.78)	0.92(0.51–1.64)	0.68(0.63–0.74)
Diabetes[Table-fn nt105] 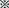	54/233	10/62	2/5	0.305	0.232	0.38	0.181	0.359	0.327
					0.64(0.30–1.34)	2.21(0.36–13.57)	3.47(0.51–23.48)	0.72(0.36–1.45)	2.41(0.40–14.71)
Obesity&	39/233	10/62	2/5	0.383	0.909	0.173	0.181	0.822	0.167
					0.96(0.45–2.04)	3.32(0.54–20.51)	3.47(0.51–23.48)	1.09(0.53–2.21)	3.35(0.55–20.56)
rs6850166(G/A)	253	43	4						
Demographic characteristics (Mean ± SD)									
Age (yr)	52.1±14.17	56.02±12.32	44.75±12.26	0.124	0.088	0.295	0.122	0.182	0.26
Age at diagnosis (yr)	45.72±14.09	48.42±12.60	42.75±12.28	0.445	0.239	0.671	0.435	0.315	0.631
Disease duration (yr)	6.37±6.56	7.60±6.37	2±1.83	0.197	0.249	0.183	0.1	0.463	0.166
BMI (kg/m^2^)	27.3±3.11	25.94±2.85	28.3±2.95	0.021	0.007	0.519	0.142	0.018	0.444
WHR	0.92±0.05	0.91±0.05	0.9±0.04	0.706	0.588	0.511	0.643	0.488	0.526
Serum Biochemistry (Mean ± SD)									
Systolic pressure (mmHg)	137.23±18.84	136.86±24.71	157.5±15	0.125	0.909	0.043	0.046	0.716	0.041
Diastolic pressure (mmHg)	88.89±12.21	84.93±11.45	97.5±9.57	0.046	0.048	0.158	0.047	0.134	0.134
Blood Glucose (mmol/L)	6.22±2.17	6.03±1.11	7.49±2.45	0.389	0.566	0.223	0.175	0.829	0.212
Uric acid (umo1/L)	513.11±141.15	536.64±122.58	405.64±270.34	0.177	0.311	0.13	0.076	0.581	0.472
Triglycerides (mmol/L)	2.26±1.69	2.19±2.03	4.03±1.6	0.127	0.806	0.045	0.045	0.759	0.044
Total cholesterol (mmol/L)	5.28±1.36	5.01±1.1	6.23±1	0.159	0.22	0.157	0.08	0.436	0.14
Urea nitrogen (mmol/L)	6.18±4.01	5.7±2.27	4.91±1.25	0.611	0.443	0.505	0.689	0.363	0.528
Creatinine (umo1/L)	93.32±40.43	91.37±36.27	73.04±24.98	0.581	0.766	0.312	0.378	0.578	0.317
Clinical characteristics									
Age at diagnosis (yr)									
<25	12/253	0	0	0.313	0.145	0.656	—	0.128	0.681
									
25–44	118/253	15/43	2/4	0.351	0.152	0.894	0.547	0.185	0.84
					0.61(0.31–1.20)	1.14(0.16–8.25)	1.87(0.24–14.61)	0.65(0.34–1.24)	1.23(0.17–8.82)
45–64	91/253	23/43	2/4	0.083	0.029	0.562	0.894	0.026	0.639
					2.05(1.07–3.93)	1.78(0.25–12.85)	0.87(0.11–6.75)	2.02(1.08–3.79)	1.60(0.22–11.49)
65–84	32/253	5/43	0	0.739	0.852	0.447	0.471	0.7	0.45
					0.91(0.33–2.48)			0.91(0.33–2.48)	
Tophi	69/253	14/43	1/4	0.768	0.476	0.919	0.756	0.515	0.893
					1.29(0.64–2.58)	0.89(0.09–8.69)	0.69(0.07–7.25)	1.25(0.64–2.45)	0.86(0.09–8.34)
Past history									
hypertension#	177/253	25/43	4/4	0.12	0.124	0.191	0.099	0.262	0.174
					0.60(0.31–1.16)	0.70(0.65–0.76)	0.58(0.45–0.75)	0.69(0.36–1.32)	0.68(0.63–0.74)
Diabetes[Table-fn nt105] 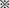	58/253	6/43	2/4	0.167	0.186	0.204	0.067	0.37	0.174
					0.55(0.22–1.36)	3.36(0.47–24.39)	6.17(0.73–52.49)	0.69(0.31–1.56)	3.63(0.50–26.24)
Obesity&	45/253	4/43	2/4	0.082	0.166	0.098	0.02	0.4	0.077
					0.47(0.16–1.39)	4.62(0.63–33.69)	9.75(1.07–89.21)	0.68(0.27–1.69)	5.04(0.69–36.65)

*The major allele was referred to as allele 1 and the minor allele as allele 2.

# Hypertension was defined as systolic blood pressure ≥140 mmHg or diastolic blood pressure ≥90 mmHg or receiving antihypertensivedrug treatment in a patient with a history of hypertension.

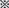
Diabetes was defined on the basis of fasting blood glucose ≥7.0 mmol/l (126 mg/dl) or non-fasting blood glucose ≥11.1 mmol/l(200 mg/dl) and/or treatment of diabetes.

& Obesity is defined as a BMI of 30 and above by World Health Organization (WHO).

Furthermore, as presented in Table 4, stratification [33] of the age of disease onset by genotypes (rs13124007 and rs6850166) did not demonstrate any different effect, except that patients carrying genotype GA at polymorphism rs6850166 accounted for more patients whose age of disease onset ranged from 45 to 64 years compared to those carrying genotype GG (P = 0.029). However, the analysis indicated no significant differences in other phenotypic disease characteristics, such as the duration of gout history, WHR, tophi, past history (including hypertension, diabetes, obesity), blood glucose, uric acid, total cholesterol, creatinine and urea nitrogen levels in plasma (all P>0.05).

### Prediction

Promoter polymorphisms might influence transcription factor binding activity; therefore, we analyzed the two promoter polymorphisms (rs13124007 and rs6850166) using AliBaba software, which can predict potential transcription factor binding sites (Figure S1). For SNP rs13124007, if C substitutes for G, a loss of a binding site for interferon regulatory factor 1 (IRF-1) occurs (Figure S1). For polymorphism rs6850166, the change of G to A did not affect any predicted transcription factor binding site (Figure S1). To explore whether there are transcription effects of the polymorphisms in the SLC2A9 promoter region, further functional studies should be performed.

## Discussion

To the best of our knowledge, our study is the first attempt to evaluate the potential association between gout and polymorphisms and haplotypes in a presumptive promoter region. We demonstrated that rs13124007 and rs6850166, located 2059 bp and 1816 bp, respectively, upstream of the start site of exon 1 of the GLUT9 gene were genetically implicated in gout in Chinese males. We found that the frequency of the C allele of rs13124007 and the A allele of rs6850166 were higher in the group with gout. Female gout patients were not included in this study because the incidence of gout in females is lower than in males. These two SNPs were in low linkage disequilibrium (*r* ^2^ = 0.56). Furthermore, haplotype analysis showed that gout patients had a significantly higher frequency of the risk haplotype CA than the protective haplotype GG. In addition, we discovered five new SNPs that were not registered in the National Center for Biotechnology Information SNP database (NCBI dbSNP). However, variants in a 1.5 kb presumptive promoter region upstream of the transcription start site of the GLUT9 gene did not influence serum uric acid levels in the Sardinia and Chianti Cohorts [17].

In 2007, a genome-wide association study found that the major locus that exerted an influence on serum urate concentration was the *SLC2A9* gene [17]. This initial study was rapidly followed by six genome-wide association (GWA) studies and other analyses, which reported that genetic variants of the SLC2A9 gene were associated with serum uric acid levels [10], [11], [16], [17], [18], [19], [20], [21], [22], [23], [24] and gout [10], [16], [25], [26], [27], [28], [29], [30] in other cohorts. To date, 14 genetic variants in *SLC2A9* (based on eight study populations) have been reported to be associated with gout in humans (Table S2). However, these genetic variants, which are mainly from exonic and intronic regions of the SLC2A9 gene, are only SNP markers and no functional studies on the effects of genetic variants have been reported. Therefore, our findings indicate that a functional region might be located in the presumptive promoter region of the GLUT9 gene.

Functionally, five groups have confirmed that human GLUT9 is a functional high-capacity/low-affinity urate transporter, by expressing SLC2A9L and SLC2A9S splice variants in Xenopus laevis oocytes [9], [10], [14], [34], [35]. Bibert S *et al.* carried out detailed transport studies and demonstrated that the mouse mGlut9a and mGlut9b splice variants were high-capacity urate transporters, with a *Km* for urate of approximately 650 µM. Transport was electrogenic and independent of the Na(+) and Cl(−) transmembrane gradients, but dependent on membrane potential [35].

In addition, the results of our study showed a significant difference in statistics between SNPs rs13124007 and rs6850166 and the status of tophus. The C allele of rs13124007 and the A allele of rs6850166 were both found to be associated with increased risk of tophus occurrence. Consistent with these findings, in a study of the risk allele of *SLC2A9*'s influence of tophus occurrence in Chinese and Solomon Island population by Hung-Pin Tu *et al.* [26], it was found that the risk allele of rs3733591 was associated with tophaceous gout in Han Chinese subjects (p = 0.0044, OR 2.01 [95% CI 1.24–3.37]) and in Solomon Islanders (p = 0.0184, OR 2.15 [95% CI 1.13–4.09]). Furthermore, the risk allele of *SLC2A9* SNPs rs3733589 and rs1014290 were both associated with increased risk of tophus occurrence in Han Chinese subjects. However, the precise molecular mechanism by which *SLC2A9* variants increase the risk of tophi development is not yet understood. Hung-Pin Tu *et al.* [26] hypothesized that tophi development is characterized by urate crystal deposition in and around the joints by chronic mononuclear and giant-cell reactions [7], [36]. SLC2A9 is an uric acid transporter which is expressed in articular cartilage [37] and peripheral leucocytes [38]. The C-allele of SNP rs3733591 might play a significant role in influencing the crystallization of urate in Chinese and Solomon Island population [26].

We presented the first detailed genotype-phenotype analysis of SLC2A9 gene variants in a Chinese population. In our study, carriers of the genotype CC for polymorphism rs13124007 and carriers of the genotype AA for polymorphism rs6850166 showed higher serum triglycerides levels compared to non-carriers. Moreover, we explored the relationship between rs6850166 and BMI values and diastolic blood pressure, and patients with the AA genotype were associated with elevated systolic pressure levels compared with the GG genotype and the heterozygous genotype. Therefore, it was of special interest that the SLC2A9 SNPs rs13124007 and rs6850166 were associated with both gout susceptibility and gout phenotype. This might be explained if the SLC2A9 gene is the common pathway that links gout with metabolic syndrome [10] and these other phenotypic disease characteristics in our study, such as serum triglycerides levels, BMI values, diastolic blood pressure, and systolic pressure levels. When further stratifying the analysis by the age of disease onset, no association between the genotypes (rs13124007 and rs6850166) and the age of disease onset was found. However, a very recent study found increasing age decreased the association between *SLC2A9* SNPs with uric acid levels in men [23].

In our study, there are several limitations that have to be considered. Firstly, we concentrated on only genetic variants, and the two polymorphisms (rs13124007 and rs6850166) are located 2059 bp and 1816 bp upstream of the *GLUT9* start site of exon 1. Therefore, further functional studies on the effect of genetic variants on transcription factor binding activity and changes at the protein level will be necessary. Secondly, when we subdivided the gout patients into tophi and non-tophi cases, we relied only on tophus status and lacked important information on the number of tophi. More detailed data analysis might further clarify the interactions between tophus number and genetic variants. Thirdly, the sample size in our research is relatively small, and all the subjects were ethnic Han Chinese, which might not be representative for other human races. Nonetheless, due to the concern that female gout patients are not included in this study and the mean age showed no significant differences in cases and controls (*P* = 0.938), age and gender, which are regarded as the two important confounding effects on association with gout, are excluded. The next obvious step is to replicate these results in other independent populations and on a much larger sample. Fourthly, the gout phenotypes are assessed retrospectively from patients' verbal reports and medical history. The data for some of phenotypic disease characteristics, such as the duration of gout history and past history, are not described exactly due to perceived lack of clinical relevance and the data do not include sufficient information on disease location, urate nephrolithiasis, metabolic syndrome, insulin resistance, cardiovascular diseases, and chronic kidney disease. Our genotype-phenotype analysis is too over-simplistic to explain the complexity of the phenomenon. Further investigation of patients' detailed clinical history will be needed.

Despite these limitations, this is the first report to indicate an association between gout and polymorphisms (rs13124007 and rs6850166) and haplotypes in the presumptive promoter region upstream of the transcription start site of the GLUT9 gene in a Chinese population. The C allele of rs13124007 and the A allele of rs6850166 might be crucial risk factors of tophus occurrence, offer the potential for further genetic investigation into the interaction between gene variants and tophi development. Furthermore, these results show the first detailed genotype-phenotype analysis of the SLC2A9 gene variants in Han Chinese. However, our findings need to be validated by other independent populations and in a much larger sample with well-designed clinical investigations and functional analyses, to shed light on potential mechanisms underlying the links between the *GLUT9* promoter polymorphisms (rs13124007 and rs6850166) and the risk of gout.

## Supporting Information

Figure S1 **Effect of the Polymorphisms rs13124007 and rs6850166 on transcription factor binding sites.** Comparison between G allele (i) and A allele (ii) in polymorphism rs13124007 and between G allele (I) and A allele (II) in polymorphism rs6850166 for the putative transcription factor binding sites by AliBaba version 2.1 software.
(TIF)

Table S1 **Primer sequences and PCR conditions used for amplification for promoter of the human GLUT9 gene.**
(DOC)

Table S2 **Assocations between SLC2A9 SNPs and gout in case-control cohorts.**
(DOC)
