# Supplementary figures and images for: Polymorphisms in the Presumptive Promoter Region of the SLC2A9 Gene Are Associated with Gout in a Chinese Male Population
Source: PLoS One. 2012 Feb 29;7(2):e24561. doi: 10.1371/journal.pone.0024561 (PMC3290627; doi:10.1371/journal.pone.0024561)

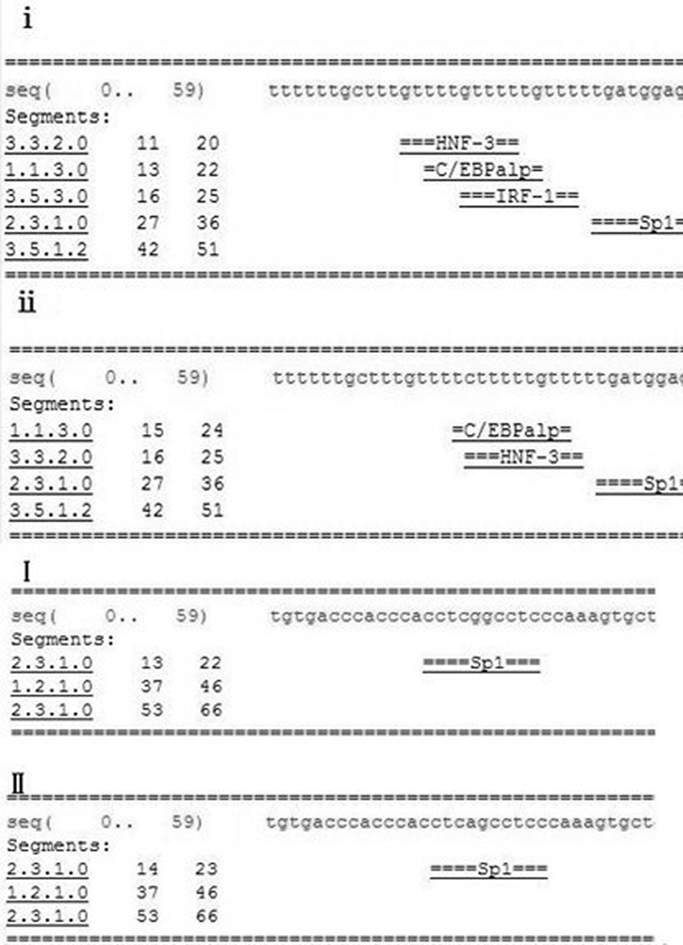

Supplement: Figure S1 — Effect of the Polymorphisms rs13124007 and rs6850166 on transcription factor binding sites. Comparison between G allele (i) and A allele (ii) in polymorphism rs13124007 and between G allele (I) and A allele (II) in polymorphism rs6850166 for the putative transcription factor binding sites by AliBaba version 2.1 software. (TIF) [file pone.0024561.s001.tif]
