# Supplementary material for: Polymorphisms in the Presumptive Promoter Region of the SLC2A9 Gene Are Associated with Gout in a Chinese Male Population
Source: PLoS One. 2012 Feb 29;7(2):e24561. doi: 10.1371/journal.pone.0024561 (PMC3290627; doi:10.1371/journal.pone.0024561)
Supplement: Table S1 — Primer sequences and PCR conditions used for amplification for promoter of the human GLUT9 gene. (DOC) [file pone.0024561.s002.doc]

| Table S1 Primer sequences and PCR conditions used for amplification for promoter of the human GLUT9 gene | | | |
| --- | --- | --- | --- |
| Amplicon | Size of PCR | Primer sequences | Annealing |
|  | product,bp | (forward, reverse) | temperature,°C |
| Promotor-1 | 566 | ATTTCCTGTTTGATTATGAC | 56.3 |
|  |  | GTGCTTACAATGATTTGC |  |
| Promotor-2 | 743 | ACGCCCGGCCATTTTTTG | 62 |
|  |  | ACGTTTCTGACCCCGGTTTT |  |
| Promotor-3 | 785 | CTGCAAATTCTATGGCCTTCTGG | 63.3 |
|  |  | GTAGCAACCGCAGAGCCACAC |  |
| Promotor-4 | 795 | CTAGGCCAGCAGTCATCAGATAC | 62 |
|  |  | GTTTTCATGCCCCTCTGGAGTC |  |
