# Supplementary material for: Polymorphisms in the Presumptive Promoter Region of the SLC2A9 Gene Are Associated with Gout in a Chinese Male Population
Source: PLoS One. 2012 Feb 29;7(2):e24561. doi: 10.1371/journal.pone.0024561 (PMC3290627; doi:10.1371/journal.pone.0024561)
Supplement: Table S2 — Assocations between SLC2A9 SNPs and gout in case-control cohorts. (DOC) [file pone.0024561.s003.doc]

| Table S2 Assocations between SLC2A9 SNPs and gout in case-control cohorts | | | | |
| --- | --- | --- | --- | --- |
| SNP marker | Region | Population | Phenotype | Reference |
| rs1014290 | Intron 3 | Scottish cohort | Gout | (10) |
|  |  | Japanese | Gout | (30) |
|  |  | Han Chinese | Gout tophi | (26) |
|  |  | Croatia | Gout nephrolithiasis | (29) |
| rs6449213 | Intron 4 | Germany | Gout | (25) |
|  |  | Scottish cohort | Gout | (10) |
|  |  | Croatia | Gout nephrolithiasis | (29) |
| rs3733589 | Synonym SNP(I139I) | Han Chinese | Gout tophi | (26) |
| rs7442295 | Intron 6 | Germany | Gout | (25) |
| rs11942223 | Intron 6 | New Zealand | Gout | (27) |
| rs6855911 | Intron 7 | Germany | Gout | (25) |
|  |  | Germany | Gout | (28) |
| rs737267 | Intron 7 | Scottish cohort | Gout | (10) |
|  |  | Croatia | Gout nephrolithiasis | (29) |
| rs734553 | Intron 7 | Germany | Gout | (28) |
| rs5028843 | Intron 7 | New Zealand | Gout | (27) |
| rs3733591 | Missense SNP (R265H) | Japanese | Gout | (30) |
|  |  | Han Chinese | Gout tophi | (26) |
|  |  | Solomon Island | Gout tophi | (26) |
| rs16890979 | Missense SNP(V253I) | New Zealand | Gout | (27) |
| rs12510549 | 5' | Germany | Gout | (25) |
|  | 5' | New Zealand | Gout | (27) |
| rs10489070 | 5' | the United States | Gout | (16) |
| rs733175 | 5’ | Croatia | Gout nephrolithiasis | (29) |
